# Role of Adult Tissue-Derived Pluripotent Stem Cells in Bone Regeneration

**DOI:** 10.1007/s12015-019-09943-x

**Published:** 2019-12-11

**Authors:** Liudmila Leppik, K. Sielatycka, D. Henrich, Z. Han, H. Wang, M. J. Eischen-Loges, K. M. C. Oliveira, M. B. Bhavsar, M. Z. Ratajczak, J. H. Barker

**Affiliations:** 1 Frankfurt Initiative for Regenerative Medicine, Experimental Orthopedics & Trauma Surgery, J.W. Goethe University, Frankfurt am Main, Germany; 2 Institute of Biology, Faculty of Exact and Natural Science, University of Szczecin, Szczecin, Poland; 3 Department of Trauma, Hand & Reconstructive Surgery, J.W. Goethe University, Frankfurt/Main, Germany; 4 Stem Cell Institute at the James Graham Brown Cancer Center, University of Louisville, Louisville, KY USA

**Keywords:** VSEL, Stem cells, Bone healing, Critical size defect model, Osteoclastogenesis, In situ hybridization

## Abstract

**Background:**

Bone marrow-derived mononuclear cells (BM-MNC) consist of a heterogeneous mix of mesenchymal stem cells (MSC), hematopoietic progenitor cells (HPC), endothelial progenitor cells (EPC), monocytes, lymphocytes and pluripotent stem cells. Whereas the importance of MSC and EPC has been well documented in bone healing and regeneration studies, the role of pluripotent stem cells is still poorly understood. In the present study we evaluated if and how Very Small Embryonic Like cells (VSEL), isolated from rat BM-MNC, contribute to bone healing.

**Methods:**

Large bone defects were made in the femurs of 38 Sprague Dawley female rats and treated with β-TCP scaffold granules seeded with male VSEL; BM-MNC, VSEL-depleted BM-MNC or scaffold alone, and bone healing was evaluated at 8 weeks post-surgery.

**Results:**

Bone healing was significantly increased in defects treated with VSEL and BM-MNC, compared to defects treated with VSEL-depleted BM-MNC. Donor cells were detected in new bone tissue, in all the defects treated with cells, and in fibrous tissue only in defects treated with VSEL-depleted BM-MNC. The number of CD68+ cells was the highest in the VSEL-depleted group, whereas the number of TRAP positive cells was the lowest in this group.

**Conclusions:**

Based on the results, we can conclude that VSEL play a role in BM-MNC induced bone formation. In our rat femur defect model, in defects treated with VSEL-depleted BM-MNC, osteoclastogenesis and bone formation were decreased, and foreign body reaction was increased.

## Introduction

Bone non-unions and large defects, due to trauma, disease, excision of tumors or congenital defects, are important clinical problems that represent a major challenge for the patients who suffer with them, the physicians who treat them and the healthcare system, responsible for their high costs. Current treatments such as callus distraction, cortical allografts, and metallic, polymeric or ceramic implants, enjoy varying degrees of success, although, autologous bone grafts are still considered to be the gold standard treatment. Despite this, drawbacks such as the need for multiple surgeries, limited amounts of graft material in overly large defects, and donor-site morbidity are major problems when autologous bone grafts are used [1, 2].

Bone Tissue Engineering (BTE) treatments, that use different combinations of osteoprogenitor cells, osteoconductive scaffolds, and growth factors hold great promise for achieving optimal healing, while at the same time eliminating many drawbacks associated with conventional treatments. For BTE applications, bone marrow-derived mononuclear cells (BM-MNC), one of several constituents contained in autologous bone grafts, are combined with osteoconductive scaffolds and growth factors and have reported encouraging outcomes in early preclinical animal studies and clinical trials [3, 4].

BM-MNC consists of a heterogeneous mix of mononuclear cells containing mesenchymal stem cells (MSC), hematopoietic progenitor cells (HPC), endothelial progenitor cells (EPC), and pluripotent stem cells [5]. Whereas the importance of MSC and EPC has been well documented in several bone healing and regeneration model systems and experimental protocols, the role of pluripotent stem cells in these treatments is still poorly understood. Adult pluripotent stem cells have been described by several groups, and depending on the group and the isolation protocol used, have been assigned different names, including, spore-like stem cells [6], multipotent adult stem cells (MASC) [7], multilineage-differentiating stress enduring (Muse) cells [8], multipotent adult progenitor cells (MAPC) [9], and very small embryonic-like stem cells (VSEL) [10, 11]. Regardless of the name used to describe these cells, they all appear to share certain unique characteristics, i.e. small size, expression of markers associated with pluripotency, and being present in very low concentrations. VSEL have been found in many adult tissues [12], have been shown to be pluripotent in vivo [13] and to stimulate new bone formation [13, 14]. The number of circulating VSEL has been shown to be increased in patients with severe myocardial and liver damage, stroke, and bowel inflammation [15–18] leading some to speculate that they may contribute to regeneration of damaged tissues [19–22].

In the present study we isolated VSEL (small size, SSEA1+, CD45-) from rat bone marrow and evaluated, if and how these cells contribute to bone healing. To determine the role VSEL play in bone healing, we treated large rat femur defects with different combinations of scaffold, VSEL and BM-MNC, and measured bone healing at 8 weeks.

### Material and Methods

All animal experiments were performed in accordance with guidelines established by our institutional animal care and oversight committee (Project No. FU/1165; Regierungspräsidium, Darmstadt, Germany), according to German law.

#### BM-MNC Isolation

Bone marrow was flushed from the bone marrow cavities of tibias and femurs of young (5–6 weeks) male Sprague Dawley (SD) rats, and the cell suspension was collected and filtered through a 70 μm strainer (BD Bioscience). Nucleated cells were obtained following lysis of red blood cells (RBCs) with 1x BD Pharm Lyse Buffer (BD Pharmingen), then washed with phosphate buffered saline (DPBS; w/o Ca^2+^, Mg^2+^; Life Technologies) and resuspended in DMEM-based medium (Sigma-Aldrich, St. Louis, MO) containing 2% fetal bovine serum (FBS) (Lonza, Basel, Switzerland).

#### VSEL Cell Isolation from BM-MNC

BM-derived VSEL were isolated from a fraction of nucleated cells isolated from BM-MNC by immune-labeling with monoclonal antibodies against CD45 (APC-CD45, Thermo Fischer), and SSEA-1 (FITC-SSEA1, BioLegend) for fluorescence activated cell sorting (FACS). Staining was performed for 30 min at 4 °C and bone marrow-derived VSEL were sorted as FSC^low^/SSC^low^/CD45^−^/SSEA-1^+^ cells using a BD-Influx Cell Sorter (Becton, Dickinson and Company, NJ, USA). During the sorting procedure the FCS/SSC was gated low and special attention was made to not gate debris and to control the back gate.

#### VSEL depletion from BM-MNC

**Fig. 1:**
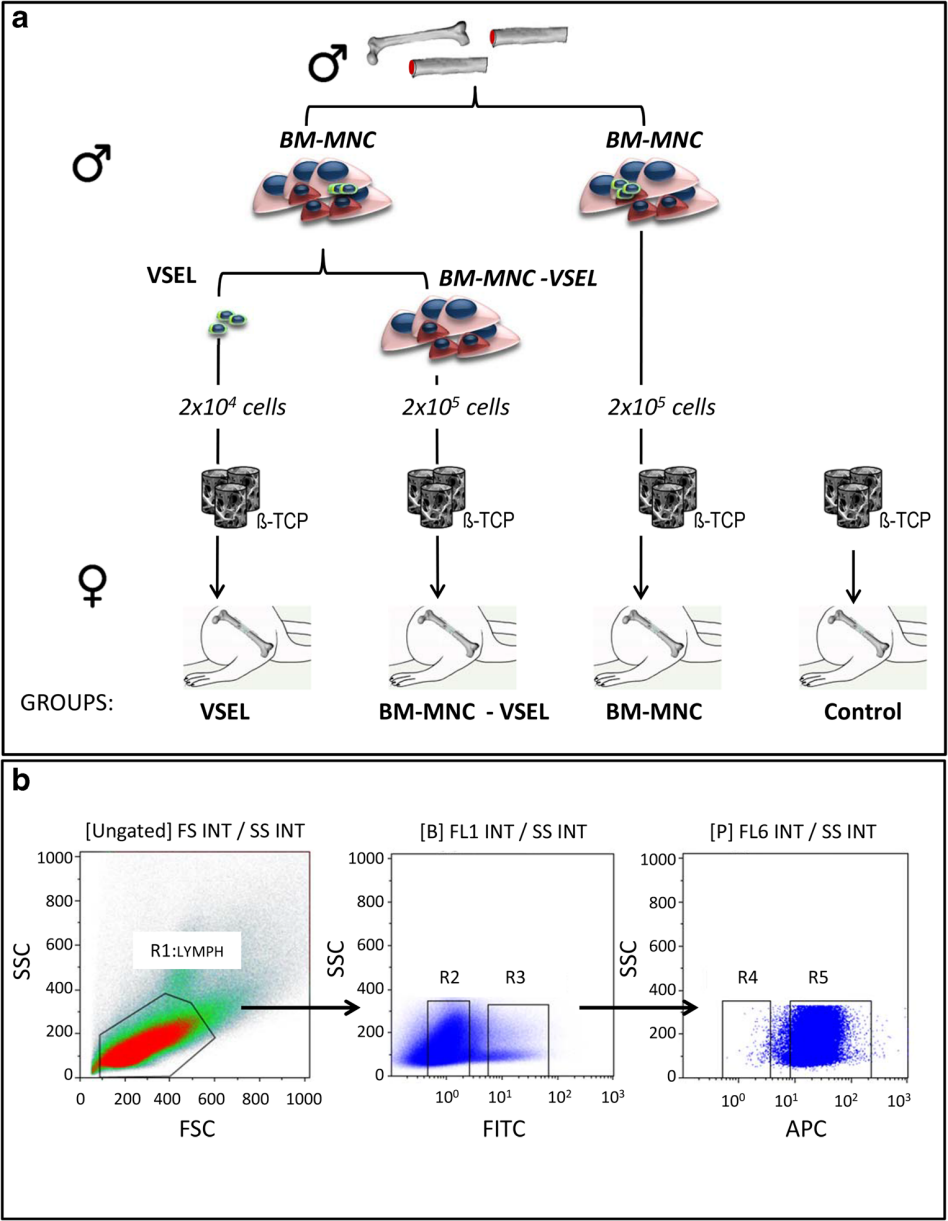
**Experimental design and VSEL isolation strategy. a** Experimental design: BM-MNC were isolated from the femurs of male SD rats and were either used to treat defects or further sorted to obtain VSEL and BM-MNC depleted of VSEL. Isolated VSEL and BM-MNC were seeded onto β-TCP scaffold granules and transplanted into female rat femur defects. Defects treated with scaffold material alone served as controls. **b** Rat BM-derived VSELs were isolated from full population of BM cells stained for CD45 (APC), and SSEA-1 (FITC). Total nucleated cells (TNCs) are visualized on dot-plot showing FSC (forward scatter) versus SSC (side scatter) signals, which are related to the size and granularity/complexity of the cell, respectively. Single cells from gate R1 are subsequently analyzed for SSEA-1 marker expression. SSEA-1+ events included in region R3 are further plotted on dot-plot showing CD45- expression versus side scattered of these cells (Region R4). Cells in region R4 were considered as VSEL and subsequently isolated using a cell sorter

BM-MNC were depleted of VSEL by similar immunostaining and collection of rest cells (except VSEL) (Fig. 1).

#### Transportation of Isolated Cells and Cell Seeding onto Scaffold Granules

After isolation, cells were collected in a sterile tube with DMEM medium and transported from Szczecin (Poland) to Frankfurt am Main (Germany) at 4 °C via TNT express overnight courier. Upon arrival in Frankfurt cell viability was confirmed using trypan blue exclusion method. 0.5 ml β-TCP granules (Chronos; Synthes, Umkirch, Germany) (0.7–1.4 mm diameter, 60% porosity, 100–500 μm pore size) were placed in 6-well plates and soaked in PBS overnight before cell seeding. 2 × 10^4^ VSEL, 2 × 10^5^ BM-MNC or BM-MNC depleted of VSEL were seeded onto β-TCP scaffold granules. All cell-seeded and non-seeded scaffolds were incubated at 37 °C, 5% CO_2_ in a humidified incubator one hour prior to being transplanted into the femur defect, and were transported to the animal facility.

#### Cell Seeded Scaffold Implantation in Rat Femur Defect

Thirty eight, nine-week-old female SD rats (Janvier Labs, Germany) were randomly allocated into four groups that received: 1) β-TCP scaffold + VSEL (*n* = 7), 2) β-TCP scaffold + BM-MNC (*n* = 12) 3) β-TCP scaffold + VSEL-depleted BM-MNC (*n* = 9), and 4) β-TCP scaffold alone (Controls; *n* = 10) (Table 1, Fig. 1). Under general anesthesia (Ketamine, 100 mg/kg and xylazine hydrochloride, 10 mg/kg, IP), the right hind limbs of rats were shaved, cleaned with antiseptic fluid and a 3 cm longitudinal dermal incision was made over the femur. The superficial fascia was incised and the tensor fascia lata, biceps femoris, and vastus lateralis muscles were elevated from the greater trochanter exposing the lateral aspect of the femur. A six-hole locking titan plate (LCP Compact Hand 1.5 Straight; DePuy Synthes, Dubendorf, Switzerland) was fixed to the lateral aspect of the femur with 2 proximal and 2 distal cortical screws (DePuy Synthes). Once the plates were fixed in place to stabilize the bone a 5 mm long defect was created on the femur shaft beneath the mid-point of the plate using a 0.22 mm gigli wire saw (RISystem, Davos, Switzerland). After the defects received their respective treatments they were irrigated with sterile saline, the fascia was re-approximated and sutured (3–0 Vicryl; Ethicon, Norderstedt, Germany) and the skin was closed with continuous intradermal sutures (4–0 Prolene; Ethicon).

**Table 1:** Experimental groups

Group	Treatment	No. of cells	No. of animals	Analysis (8 weeks)
VSEL	β-TCP + VSEL	2 × 10^4^	7	Histology, IHC, CISH
BM-MNC	β-TCP + BM-MNC	2 × 10^5^	12	Histology, IHC, CISH
VSEL-depleted BM-MNC	β-TCP + VSEL-depleted BM-MNC	2 × 10^5^	9	Histology, IHC, CISH
Control	β-TCP alone	–	10	Histology, IHC, CISH

#### Histological Assessment of New Bone Formation

Bone healing was assessed by histological measurements of the defect, performed at 8 weeks post-surgery. Animals were euthanized using CO_2_ inhalation and their femurs were dissected free and examined macro- and microscopically for signs of infection or tumors. Plates and screws were removed and femurs were fixed in Zinc-Formal-Fixx (Thermo Fischer Scientific, Waltham, USA) for 24 h, decalcified in 10% EDTA/TRIS-HCl (pH 7.4) for 14 days, and embedded in paraffin for subsequent histomorphometric analysis. To assess healing, tissue sections (5-7 μm) were taken parallel to the long axis of the femur and stained with Alcian Blue-Orange G-Hematoxylin-Eosin according to the protocol published in [23]. Images of the sections were captured using light microscopy (Ti-E, Nikon GmbH, Dusseldorf, Germany) and quantitative evaluations were performed using image analysis software (NIS-Elements software, Nikon GmbH) as described before [24], with some modifications. Briefly, the original bone defect area was determined and measured in μm^2^ using the “polygon-area measurement tool” of the image analysis software. The area of newly formed bone in the original bone defect a) protruding from the cut bone ends, or b) located in the center of the defect (on the scaffold granules), were outlined and measured with the same tool. The dimensions of each area of newly formed bone (a and b) were then normalized to the size of the original defect area. A minimum of three slides per animal, and the mean value of 5 animals per group, were used for subsequent statistical analysis.

#### Y Chromosome Probe and In Situ Hybridization

A digoxygenin (DIG)-labeled 200-bp probe of rat Y chromosome was created using DIG-High Prime DNA Labeling and Detection Starter Kit I (Sigma-Aldrich, Munich, Germany) according to the manufacturer’s protocol. Briefly, genomic DNA, isolated with DNeasy Blood&Tissue Kit (Qiagen, Hilden, Germany) from male rat tissues was used as a template to generate a CISH probe. The probe template (254 bp) was amplified with *SRY1* gene-specific primers (forward TTTATGGTGTGGTCCCGTGG and reverse GTTGAGGCAACTTCACGCTG; Sigma-Aldrich, Germany) and after confirming successful amplification, the PCR product was purified with a QIAquick PCR purification kit (Qiagen). 600 ng of purified PCR product was DIG- labeled overnight at 37 °C and labeling efficiency was estimated with dot blot hybridization according to the manufacturer’s manual. Y-chromosome in situ hybridization was carried out as follows: Paraffin embedded tissue sections were deparaffinized and rehydrated in decreasing solutions of ethanol. Proteinase K (10 μg/ml; CarlRoth, Karlsruhe, Germany) was applied for 10 min at room temperature, washed and endogenous alkaline phosphatase (AP) was deactivated by incubation of the tissue sections in ice-cold 20% acetic acid for 20 s. After rinsing in water, the tissue sections were dehydrated in increasing ethanol solutions (70%, 90%, and 100%) and air-dried. For each 8 sections, 2 μl of DIG-labeled probe was mixed with 10 μl of hybridization buffer (50% Formamide, 1 M NaCl, 25 mM EDTA, 50 mM Tris-HCl, 25 mM NaH_2_PO_4_, 25 mM Na_2_HPO_4_, 1x Denhardt’s solution, 10% Dextran sulphate, 20kU/ml Heparin and 10% SDS, all purchased from Sigma-Aldrich), denaturated for 10 min at 95 °C and immediately cooled on ice. For hybridization, denaturated probe was mixed with 400 μl of hybridization buffer and 50 μl of hybridization/probe mix was pippeted over each section and sealed with silicone Hybrislip cover glasses (Sigma-Aldrich) and rubber cement (Marabu GmbH, Tamm, Germany). Tissues were denaturated for 10 min at 70 °C, cooled on ice and finally incubated at 37 °C overnight in a humidified chamber. Subsequently, cover glasses were removed and sections were washed twice with 2x SSC buffer, twice with 0.2xSSC buffer and once with 1xMABT buffer, all at room temperature. After washing, the sections were blocked (2%BSA in MAB buffer) for 1 h and incubated with AP-conjugated anti-DIG antibody (1:250 in blocking solution) for 1 h, all at room temperature. After washing with MABT buffer and 10 min incubation in pre-staining buffer (100 mM Tris pH 9.5, 100 mM NaCl, 10 mM MgCl_2_) sections were covered with 70 μl nitro blue tetrazolium and 5-bromo-4-chloro-3-indolylphosphate substrate solution. After 3 h the incubated sections were washed with tap water, background staining was performed with FastRed (Sigma-Aldrich) solution for 3 min and sections were mounted with glycerin gelatin (Karl Roth) for microscopy evaluation. Stained sections were analyzed at high (20x) magnification with a light microscope, for the presence of positive stained cells.

#### CD68 Immunohistochemistry Analysis

Tissue sections were deparaffinized, rehydrated and trypsin antigen retrieval was performed before staining with antibodies. Samples were incubated with mouse anti-rat CD68 primary antibodies (1:100, MCA341GA; BIO-RAD Laboratories; Feldkirchen, Germany) at 4 °C overnight. For signal detection, an EnVision + System-HRP (AEC) kit (Dako, Glostrup, Denmark) was used. Finally, a counterstain with hematoxylin was performed. An Isotype identical (IgG1) non-specific mouse antibody served as a negative control (eBioscience, San Diego, USA). Three slides per animal were analyzed using light microscopy (at 10x) (Ti-E, Nikon) and image analysis software (NIS-Elements 4.4, Nikon). Positive CD68- and hematoxylin-stained cells were thresholded in the defect area (ImageJ software, [25]), and for each defect, the area with CD68-positive cells was normalized to the total (hematoxylin-stained) area of cells, to obtain the ratio of CD68 cells in each defect. The mean value of 5 animals per group was used for subsequent statistical analysis.

#### TRAP Staining for Osteoclasts

TRAP staining solution was prepared as follows; 1 ml of Naphtol AS-MX Phosphate Substract Mix (2% in 2-Ethoxyethanol; Sigma-Aldrich) was mixed with 120 mg Fast Red Violet LB Salt in 200 ml of TRAP basic incubation medium (0,1 M Sodium Acetate; 0,05 M Sodium L-Tatrate dibasic dehydrate; pH = 4,7) all purchased from Sigma-Aldrich). Tissue sections were deparaffinized, rehydrated and stained with pre-warmed (37 °C) TRAP staining solution for 45 min at 37 °C. After staining tissue slides were washed with distillated water and mounted with glycerol-based mounting medium (Carl Roth). For quantification, three 400 × 300 μM regions of interest (ROI) located at the left, center, and right of the defect were selected and analyzed at 20x magnification. Positive TRAP- stained cells were thresholded, and for each ROI the area with TRAP-positive cells was normalized to the total tissue area to obtain the ratio of TRAP-positive cells. The mean value of the 3 ROI was calculated for each animal and the values of 3–5 animals, per group were used for subsequent statistical analysis.

#### Cytokine Expression Profile

Screening for cytokines differentially expressed in VSEL and VSEL-depleted BM-MNC was performed with rat Cytokine Antibody Array (ab133991, Abcam, Berlin, Germany) according to the manufacturer’s instructions. Briefly, 100 μg of each protein lysate were hybridized to the array membrane. A biotin-conjugated secondary antibody was used and cytokines were detected by HRP-conjugated streptavidin. Chemiluminescence was detected with a ChemiDoc XRS+ System (BioRad) and densitometry was performed using ImageJ software. Relative levels of expression were calculated as the average of the sum of signal integrated density for each marker of interest minus the average of the sum of the integrated densities of the corresponding blank control spots. Normalization was performed by defining one array (BM-MNC) as the reference to which the other arrays were normalized from the average of the sum of the signal integrated density belonging to the positive control spots. The mean value of the technical duplicates was calculated and used for subsequent statistical analysis. Data are shown as a fold difference against expression in BM-MNC. Analysis was repeated twice with two different vials of sorted cells.

#### Statistical Analysis

For all parameters analyzed, a minimum of five animals per group were used. For bone formation measurements, CD68+ cells and TRAP+ cells quantification results are presented as box-plots of the median in the figures, 25%, and 75% quartiles (M (25%q/75%q). Nonparametric Kruskal–Wallis test and multiple Conover-Iman test were consequently used, and a Bonferroni-Holm corrected *p* < 0.05 was used to indicate statistical significance. The cytokine expression data are presented as mean + SD and significance level was set at *p* < 0.05. Statistics were calculated using the software *Bias* 10.03 (Epsilon-Verlag, Darmstadt, Germany).

## Results

### Bone Healing

**Fig. 2:**
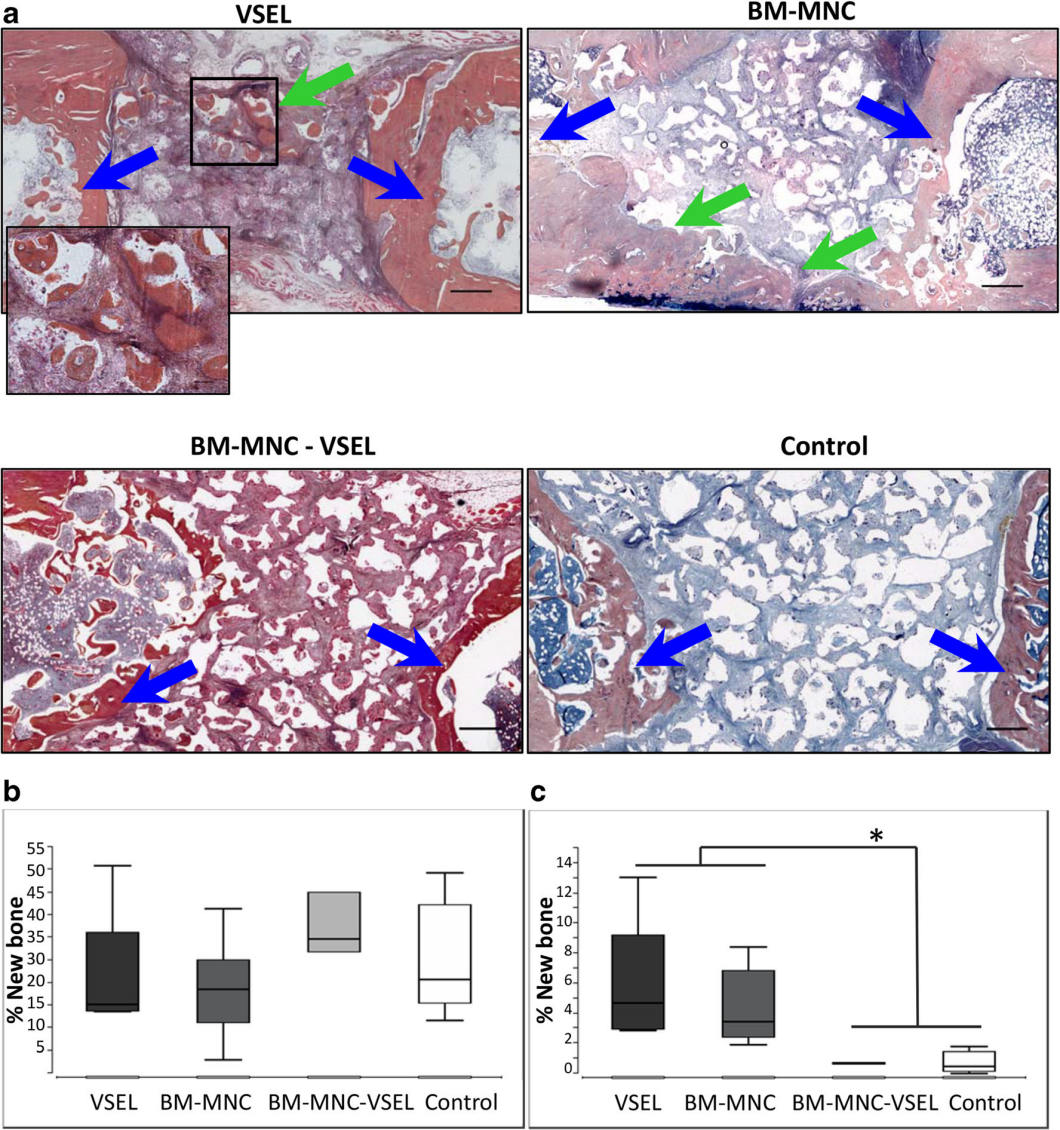
**Histological sections of femur defects. a** Defects stained with Alcian Blue, Orange-G and Hematoxylin, 8 weeks after defect creation and treatment with VSEL, BM-MNC, VSEL-depleted BM-MNC, and controls (scaffold alone). Blue arrows indicate defect margins and green arrows indicate islands of bone formation seen in defects treated with BM-MNC and VSEL alone. Defects treated with VSEL-depleted BM-MNC and controls contained mostly fibrous tissue. (Scale bar = 500 μM; high resolution image scale bar = 100 μM). **b** Graph shows the amount of newly formed bone originating from the bone cut ends/periosteum; **c** Graph shows the amount of newly formed bone originating from cell-seeded scaffold granules. Significantly less new bone formation was detected in defects treated with VSEL-depleted BM-MNC and controls (*, *p* < 0.05)

At 8 weeks none of the defects, in any of the groups were completely healed, i.e. complete bridging of the defect with bony tissue was not achieved (Fig. 2a). Detailed histological analysis near the cut ends of the bone in the VSEL-depleted group revealed large amounts of newly formed bone originating from the periosteum; however, the difference compared to the other groups was not significant. At the same time, the amount of new bone originating from cell-seeded scaffold granules, in the middle of the defect, was similar in defects treated with BM-MNC and VSEL alone, and was significantly lower in those treated with VSEL-depleted BM-MNC and controls (scaffold alone) (Fig. 2b).

### Donor Cell Detection in Defect Tissues

**Fig. 3:**
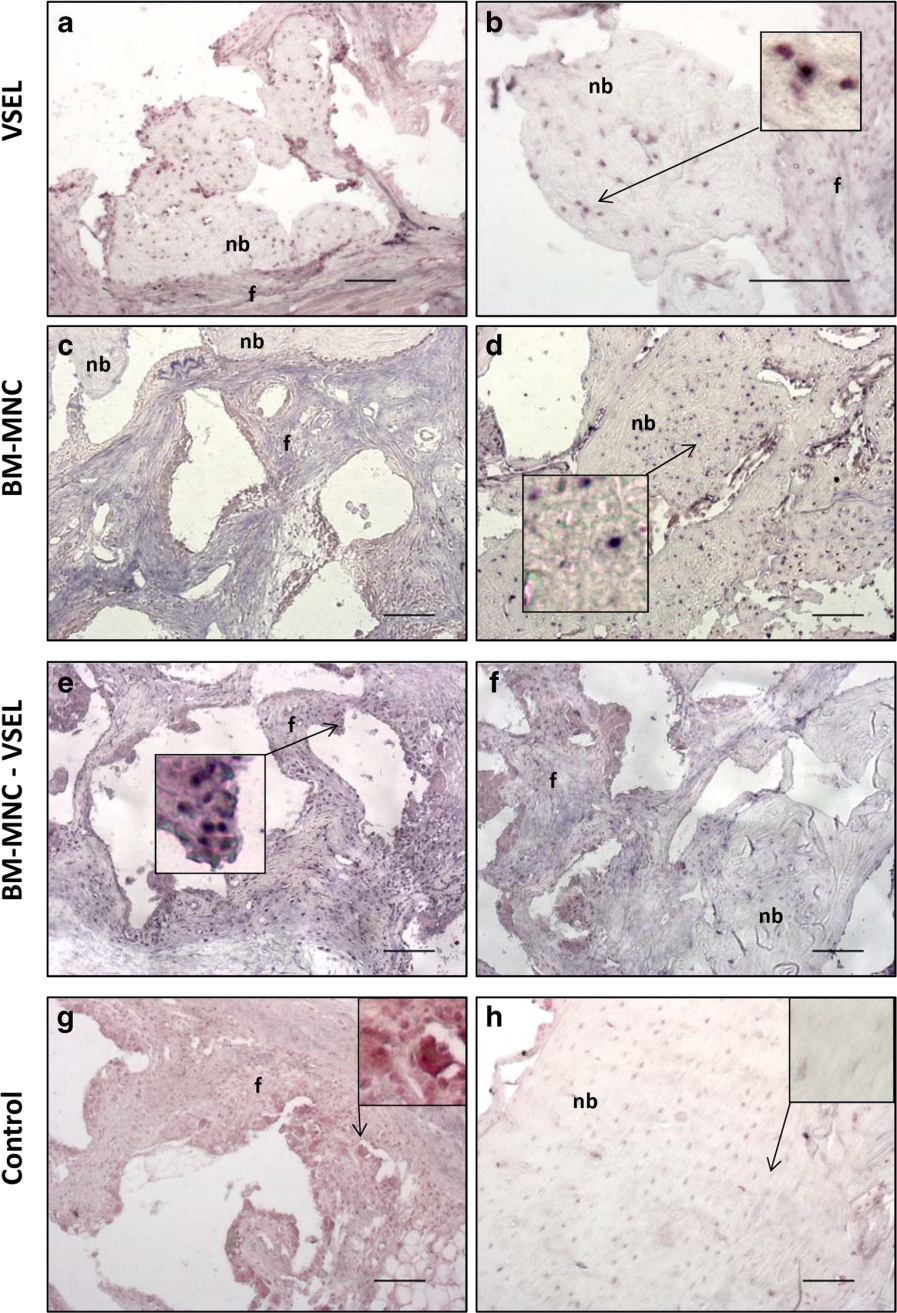
**Detection of transplanted donor cells after an ISH with digoxigenin-labeled*SRY1*- probe in defect tissue, 8 weeks after defect creation and treatment. a**–**h** Sections were hybridized with *SRY-1* probe and counterstained with nuclear Fast Red solution. The presence of male cells (dark violet/black nuclei) in newly formed bone tissue was shown in the VSEL (**a**, **b**), BM-MNC (**d**), and VSEL-depleted BM-MNC (**f**) treated groups. Donor (male) cells were also detected in fibrous tissue (f) in VSEL-depleted BM-MNC treated defects (**e**). Control samples, containing only female host cells, stained negatively (**g**, **h**). (nb- new bone; f- fibrous tissue; 20x magnification, Scale bar = 100 μM)

To identify donor cells transplanted from (male) rats, defect tissues were hybridized with a DIG-labeled probe specific for Y – chromosome *SRY1* gene (Fig. 3). No positive staining was detected in control defects where no donor cells were used for treatment. In all experimental groups positively stained donor cells were detected in newly formed bone tissue (Fig. 3a, b, d, f). In the VSEL-depleted defects positively stained donor cells were also found in fibrous tissue (Fig. 3e).

### Immune Reaction

**Fig. 4:**
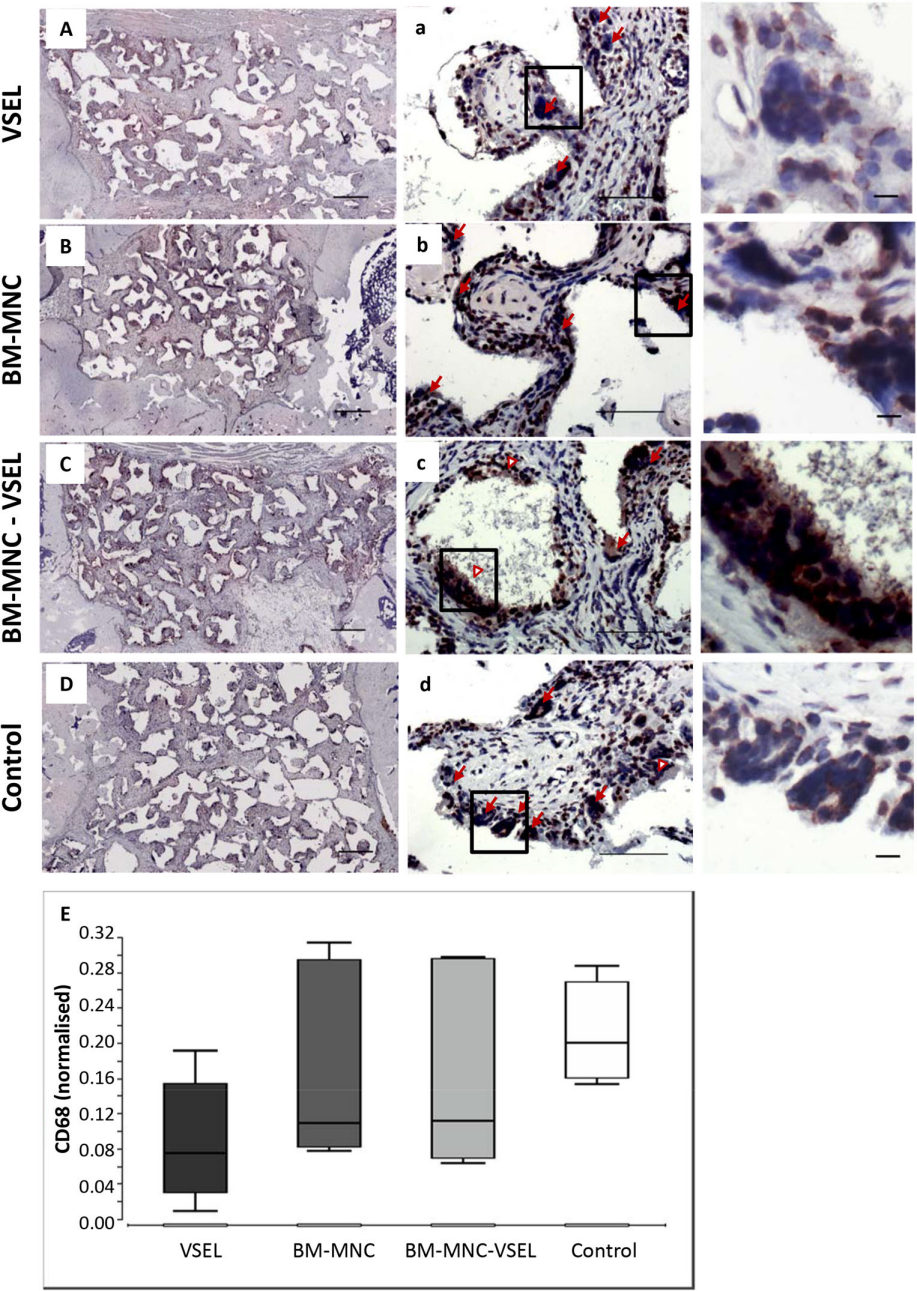
**Immunohistochemistry analysis of CD68-positive cells in defect tissue, 8 weeks after defect creation and treatment. A**–**D** Representative images of immunohistochemistry staining showing the presence of CD 68+ cells in defects treated with VSEL (**A**, **a**); BM-MNC (**B**, **b**); VSEL-depleted BM-MNC (**C**, **c**) and scaffold alone (**D**, **d**). Red closed arrows show multinuclear cells with horse-shoe shaped nuclei; red open arrows indicate giant multinucleated cells. Superimposed black squares delineate magnified area in the right panel. **A**–**D** 4x, Scale bar = 500 μM; **a**–**d** 20x, Scale bar = 100 μM. High magnification of multinucleated giant cells, Scale bar = 10 μM. **E** Ratio of CD68 positive cells in the defect area for all groups

To determine if the immune reaction to the transplanted cell-seeded scaffold granules differed among groups, tissue sections were stained with anti-CD68 antibody (Fig. 4). Positively stained cells (monocyte lineages and macrophages) were detected in all groups, however the number and the morphology of the cells differed among the groups. In the VSEL group the number of positively stained cells in the defects was lower than in the other groups (Fig. 4A, E). The maximum number of CD68 positive cells was detected in the control (scaffold alone) defects. Detailed analysis revealed that the morphology of CD68-positive cells, surrounding scaffold in the VSEL-depleted samples, differed from the morphology of CD68+ cells in the other groups. In the VSEL-depleted group these cells appeared larger in size and contained more nuclei, whereas in the other groups multinuclear cells were smaller in size and had horse-shoe shaped nuclei (Fig. 4, high magnification).

### Osteoclastogenesis

**Fig. 5:**
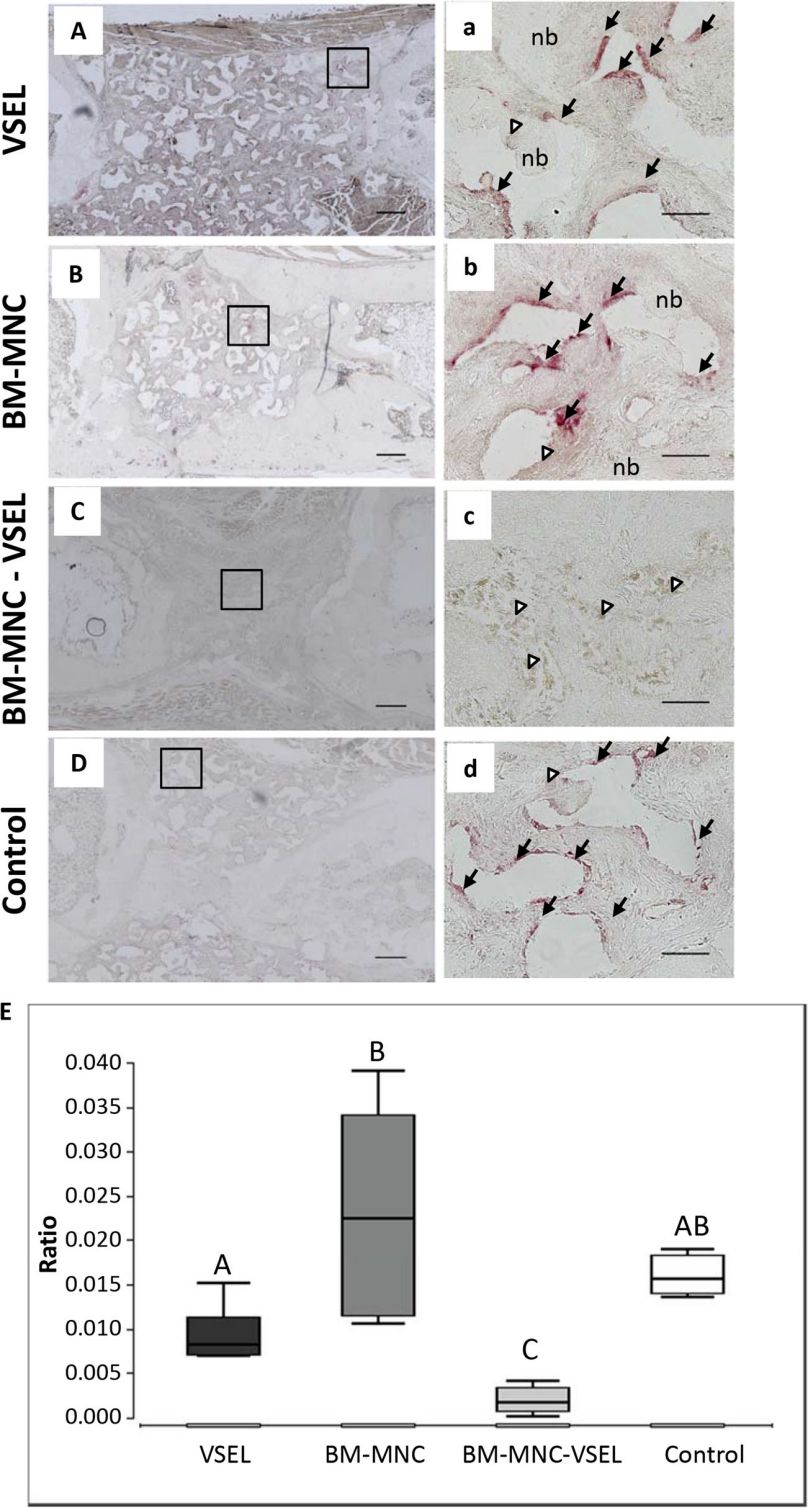
**Osteoclastogenesis. A**–**D** Representative sections of defect tissue stained for the osteoclast marker, Tartrate Resistant Acid Phosphatase (TRAP). TRAP - positive cells (black arrow) are seen in the VSEL (**a**), BM-MNC (**b**) and control (**d**) groups, whereas in the VSEL-depleted group (**c**) the majority of multinucleated cells were TRAP-negative (white triangle); **A**–**D** 4x, Scale bar = 500 μM; (**a**–**d**) 20x, Scale bar = 100 μM. **E** Graph shows quantification of TRAP positive cells in the different groups. The lowest number of TRAP positive cells was detected in the VSEL-depleted group. Different letters on bars indicate significant (*p* < 0.05) differences between groups

Significant differences between groups were detected, after TRAP staining for osteoclasts (Fig. 5). The number of TRAP positive cells in the VSEL, BM-MNC and control groups was significantly higher than in the VSEL-depleted group, with the maximum numbers observed in the BM-MNC group. The number of TRAP-positive cells in the VSEL group was significantly (*p* < 0.05) lower than in the BM-MNC and control groups, however it was significantly (*p* < 0.05) higher than in the VSEL-depleted group. The majority of multinucleated giant cells in the VSEL-depleted group were TRAP negative (Fig. 5C, c). No significant difference was detected between the BM-MNC and control groups.

### VSEL, BM-MNC and VSEL-Depleted BM-MNC Cytokine Expression Analysis

**Fig. 6:**
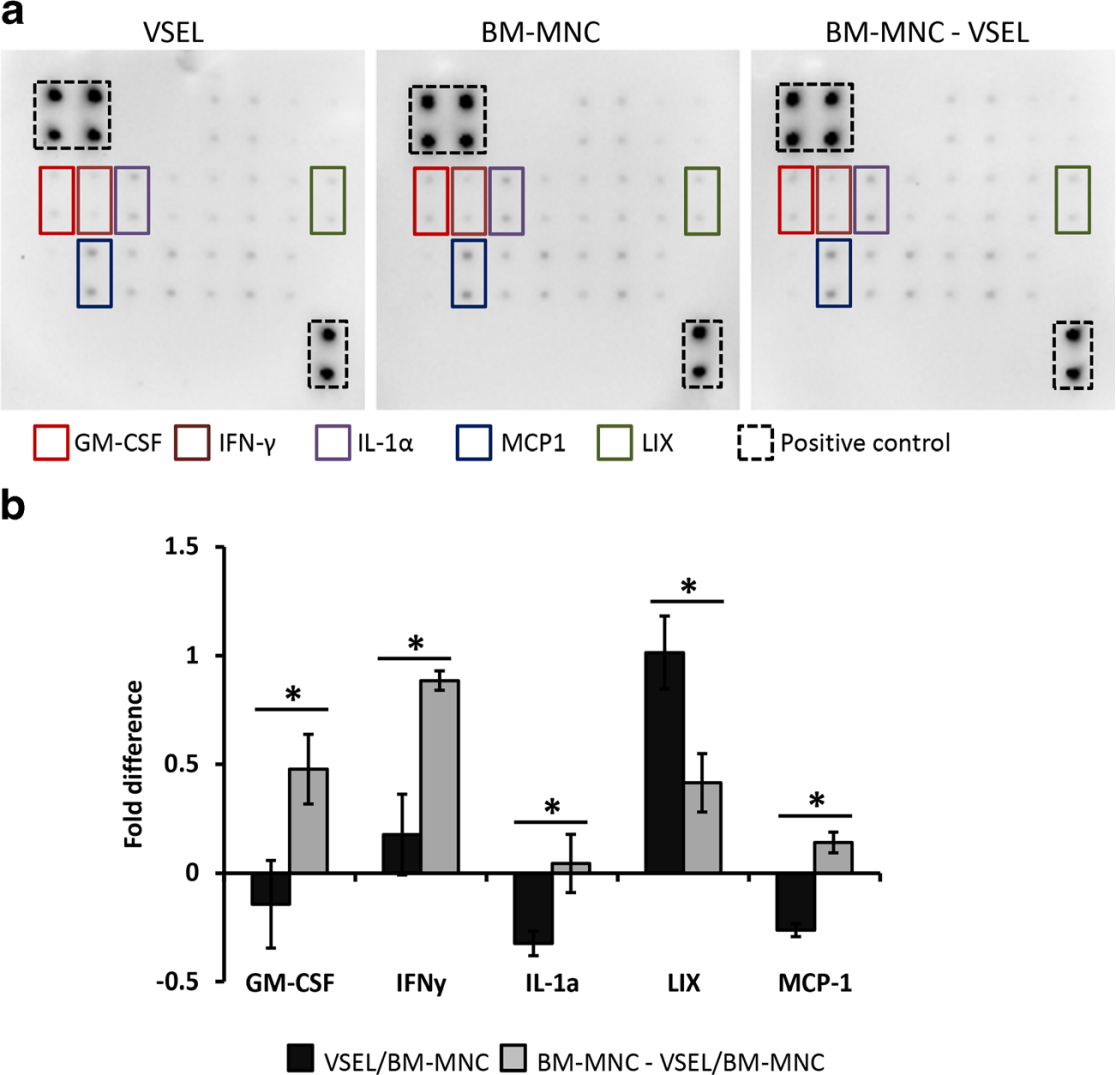
**Cytokine expression in VSEL, BM-MNC and VSEL-depleted BM-MNC. a** Expression of cytokines in VSEL, BM-MNC and VSEL-depleted BM-MNC. **b** Expression of GM-CSF, IFN-γ, IL-1α and MCP-1 was significantly downregulated and LIX significantly upregulated in VSEL compared to VSEL-depleted BM-MNC. Data represents fold difference compared to the BM-MNC cells and is expressed as the two-dot Mean ± SD. (*) *P* < 0.05

In order to compare the “immunity “of the VSEL, BM-MNC and VSEL-depleted BM-MNC, at the protein level, we performed cytokine expression array on these cells (Fig. 6). Results showed that whereas expression of most of the cytokines analyzed was slightly lower in the VSEL compared to the VSEL-depleted BM-MNC*,* expression of granulocyte-macrophage colony-stimulating factor (GM-CSF), interferon gamma (IFNγ), interleukin 1 alpha (IL-1α), and monocyte chemoattractant protein-1 (MCP-1; CCL2) were significantly (*p* < 0.05) lower, and the expression of lipopolysaccharide-inducible CXC chemokine (LIX; CXCL5) was significantly (*p* < 0.05) higher in VSEL compared to VSEL-depleted BM-MNC (Fig. 6b).

## Discussion

In previous experiments we treated rat femur large defects with different combinations of purified MSC and EPC seeded onto scaffold material, and observed significantly improved healing [26–28]. In subsequent experiments using the same femur defect model we replaced MSC and EPC with BM-MNC and observed comparable positive bone healing [29]. Considering that the concentration of MSC and EPC is very low in BM-MNC, we hypothesized that another cell fractions, besides MSC and EPC, might be involved in the observed positive healing effect [30]. The presence and osteogenic [14], hepatogenic [31] and vasculogenic [32] effects of adult pluripotent stem cells in BM-MNC identified as VSEL have been demonstrated in in vivo models by others. However, the role, and even the very existence of these cells is the focus of heated debate in the scientific literature [33, 34]. The raised controversy has been carefully addressed [35, 36] and currently more than 25 independent laboratories confirmed presence of these cells in adult murine, rat and human tissues [37]. Currently developed ex vivo expansion strategies will facilitate clinical applications of VSEL [38, 39].

In the present study we evaluated the contribution of VSEL, isolated from rat BM-MNC, to healing of large bone defects in our rat femur model. We compared healing of rat femur defects, treated with VSEL, BM-MNC, and VSEL-depleted BM-MNC plus β-TCP scaffold, and with scaffold alone, at 8 weeks after defect creation and treatment. Our results showed that VSEL contribute significantly to bone healing. Specifically, a concentration of 2 × 10^4^ VSEL alone stimulated the same amount of new bone formation, as 2 × 10^5^ of BM-MNC. This result correlates with previous findings reported by Havens et al., who demonstrated that treatment with comparable numbers of VSEL in a mouse cranial defect model resulted in significant bone healing [14]. These findings of higher regenerative capacity of less mature (SSEA-1+) VSEL in comparison to more mature cells (BM-MNC) is also supported by results from Richart, et al., who showed a similar correlation between cell maturity and regenerative capacity in a critical limb ischemia model, where embryonic stem cell (ESC)-derived SSEA-1+ cells and their progeny were compared [40]. This was reinforced in the present results in which we showed that by removing VSEL from BM-MNC healing was significantly decreased, to levels comparable to defects treated with scaffold alone (controls). The importance of VSEL for BM-MNC - induced osteogenesis, demonstrated in this study by depletion of VSEL from BM-MNC, suggests that VSEL could be involved in other BM-MNC- regenerative functions, like regeneration of infarcted myocardium [41]. The discrepancy between previous study results, showing that BM-MNC, but not HSC alone improve cardiac function [41–44], could be resolved in similar experiments using this type of depletion strategy. Despite these positive findings, the amount of healing we observed in the present experiments was less than in previous experiments. In previous experiments using the same femur defect model, treated with different combinations of MSC, EPC and scaffold we observed a greater degree of bone bridging in the defect at 8 weeks. In contrast, in the present experiments, we found that none of the defects, in any of the groups, were fully bridged with newly formed bone at 8 weeks. This might be explained by the low number of transplanted cells (2 × 10^4^ VSEL and 2 × 10^5^ BM-MNCs/depleted BM-MNCs) we used to treat the defects in the present experiments, compared to previous protocols in which defects were treated with one hundred times more cells. We used this low number of VSEL in these experiments to simulate the previously cited study by Havens, et al. in which the same amount of VSEL was found to be sufficient to provide significant bone healing in their mouse cranial defect model [14]. In addition, we used this low number of VSEL due to technical limitations, i.e. since VSEL are found in very low concentrations it was difficult to harvest large numbers of cells. To date, it has not been possible to expand rat VSEL in vitro [45, 46], therefore we could only use the limited number of cells we were able to harvest from donor rats. Another possible reason for the less amount of healing observed in these experiments could have been the time elapsed between harvesting and transplanting the cells into the femur defect. Due to logistical constraints the cells were placed in the defect one day after being isolated. While we did not detect a significant decrease in cell viability between harvesting and transplanting the cells, this delay might have affected cell activity [47, 48]. Finally, in order to identify donor cells in the defect we used male VSEL in female recipient defects. Others have described lesser amounts of bone healing in female models [49].

It is important to note a few potential limitations in this study. Due to the lack of availability of additional rat VSEL specific markers, the VSEL sorting strategy in this study was limited. Therefore the possibility of the presence of minor contamination with other cell types could not be excluded. If present, this contamination most likely did not interfere with the observed VSEL-induced osteogenic effect, however in future studies it would be important to develop a high-efficiency and high-yield rat VSEL- sorting protocol and reproduce the present study with a guaranteed completely homogenous VSEL population.

Using in situ hybridization for the male specific gene, *SRY1*, we observed donor (male) cells in newly formed bone in the defect in all three experimental groups, showing that transplanted “donor” cells were present and likely played a role in the observed new bone formation. Since transplanted stem cells have been difficult to detect in bone samples several days/weeks after transplantation, it is generally thought that these cells play an in-direct role in bone healing, through expression of paracrine molecules [50, 51]. That said, there are a few studies in which transplanted cells have been detected in defects at later timepoints [52]. These conflicting observations between studies could be explained by numerous reasons - ex vivo expansion of cells, initial cell dosage, type of scaffold, type of host animal, method of transplanted cell detection and others. Interestingly, in our defect tissues, treated with VSEL-depleted BM-MNC, we found male cells, not only in newly formed bone, but also in newly formed fibrous tissue. This finding suggests that in addition to playing a role in osteogenesis, VSEL may also exert paracrine effects on other cells.

Little is known about if and how VSEL interact with other cells and influence regeneration. It is well known, that immune and particularly foreign body reactions to transplanted cells/scaffolds play an important role in healing in bone tissue engineering treatments. Our measurements of CD68+ and TRAP+ cells in the defect tissue showed that depleting VSEL from BM-MNC resulted in a significant change in the reaction to the transplanted cell-seeded scaffold granules. Whereas in the other 3 groups the scaffold granules were surrounded by osteoclasts (CD68+, TRAP+, multinucleated cells with horse-shoe shaped nuclei), in the VSEL depleted group scaffold granules were surrounded by foreign body giant cells (CD68+, TRAP-, multinucleated cells) [53]. It was recently proposed, that in bone healing, osteoclasts and foreign body giant cells may play opposite roles in their reaction to transplants. Whereas osteoclasts dissolve scaffold granules, leading to osteointegration and bone formation, giant cells form fibrous capsules around the transplanted material, thus preventing it’s integration and bone formation [53]. This could explain the differences we observed in new bone formation between the defects treated with, and without VSEL.

It has been shown previously that bone marrow-derived MSC are able to modulate foreign body reaction and interact with host immune cells during MSC-mediated bone formation [54] by attracting circulating hematopoietic stem cells and prompting their differentiation into M1 macrophages and osteoclasts [55]. A similar mechanism could be hypothesized for VSEL. However additional studies need to be done in order to determine why removing VSEL from BM-MNC reduces bone healing. Our results of cytokine expression analysis in VSEL and VSEL-depleted BM-MNC provide some hints for answering this question. Increased expression of IL-1α, GM-CSF and MCP1 cytokines in VSEL-depleted BM-MNC could be responsible for the observed enhanced foreign body reaction, as these cytokines are known to be involved in pro-inflammatory gene transcription and fibrosis [56], monocytes and macrophages attraction [57], foreign body giant cells formation [58] and macrophage-dependent biomaterial fibrosis [59]. Decreased expression of CXCL5 cytokine in the VSEL-depleted BM-MNC, and increased expression of IFNγ may be responsible for the observed reduced new bone formation [60], as these cytokines have been shown to play an important role in bone remodeling, wound healing, angiogenesis [61] and in maintenance of hematopoietic stem cells [62].

## Conclusion

Based on the results presented in this study, we can conclude that VSEL do play a role in BM-MNC induced bone formation. In our rat femur defect model, in defects treated with VSEL-depleted BM-MNC, osteoclastogenesis and bone formation were decreased, and foreign body reaction was increased.
